# Nociception Coma Scale—Revised with Personalized Painful Stimulus Versus Standard Stimulation in Persons with Disorders of Consciousness: An International Multicenter Study

**DOI:** 10.3390/jcm13185528

**Published:** 2024-09-18

**Authors:** Rita Formisano, Marta Aloisi, Giulia Ferri, Sara Schiattone, Anna Estraneo, Alfonso Magliacano, Enrique Noé, Maria Dolores Navarro Pérez, Bahia Hakiki, Anna Maria Romoli, Erik Bertoletti, Gloria Leonardi, Aurore Thibaut, Charlotte Martial, Olivia Gosseries, Marie Brisbois, Nicolas Lejeune, Myrtha O’Valle, Joan Ferri, Anne Frédérick, Nathan Zasler, Caroline Schnakers, Marco Iosa

**Affiliations:** 1 Santa Lucia Foundation, Scientific Institute for Research and Health Care, 00179 Rome, RM, Italy; g.ferri@hsantalucia.it (G.F.); s.schiattone@hsantalucia.it (S.S.); m.iosa@hsantalucia.it (M.I.); 2 Don Carlo Gnocchi Foundation, Scientific Institute for Research and Health Care, 50143 Firenze, FI, Italy; aestraneo@dongnocchi.it (A.E.); amagliacano@dongnocchi.it (A.M.); hakiki.bahia@gmail.com (B.H.); annamariaromoli@gmail.com (A.M.R.); 3 IRENEA Neurological Rehabilitation Institute, Fundación Hospitales Vithas, 46011 Valencia, Spain; enoe@comv.es (E.N.); loles.navarro@irenea.es (M.D.N.P.); myrtha@neurorhb.com (M.O.); joan.ferri@irenea.es (J.F.); 4 Department of Experimental and Clinical Medicine, University of Florence, 50139 Firenze, FI, Italy; 5 Neurological and Internal Medicine Service “Santa Viola”, Hospital—Consorzio Colibrì, 40133 Bologna, BO, Italy; erikbertoletti@santaviola.it (E.B.); leonardigloria.psy@gmail.com (G.L.); 6 Coma Science Group, GIGA-Consciousness, University of Liège, 4000 Liège, Belgium; athibaut@uliege.be (A.T.); cmartial@uliege.be (C.M.); ogosseries@uliege.be (O.G.); marie.brisbois@ulb.be (M.B.); nicolas.lejeune@uliege.be (N.L.); 7 Centre du Cerveau2, University Hospital of Liège, 4000 Liège, Belgium; 8 William Lennox Neurological Hospital, 1340 Ottignies, Belgium; anne.frederick@chnwl.be; 9 Concussion Care Centre of Virginia, Ltd. and Tree of Life Services, Inc., Henrico, VA 23233, USA; nzasler@cccv-ltd.com; 10 Department of Physical Medicine and Rehabilitation, Virginia Commonwealth University, Richmond, VA 23284, USA; 11 Department of Physical Medicine and Rehabilitation, University of Virginia, Charlottesville, VA 22903, USA; 12 Research Institute, Casa Colina Hospital and Centers for Healthcare, Pomona, CA 91767, USA; cschnakers@casacolina.org; 13 Department of Psychology, University Sapienza of Rome, 00185 Rome, RM, Italy

**Keywords:** disorders of consciousness, nociception coma scale, coma recovery scale—revised

## Abstract

**Background/Objectives:** Persons with disorders of consciousness (DoCs) may perceive pain without being able to communicate their discomfort. The Nociception Coma Scale (NCS) and its revised form (NCS-R) have been proposed to assess nociception in persons with DoCs. The main aim of this international multicenter study was to confirm (or not) our preliminary results and compare the NCS-R scores of standard stimulus (NCS-R-SS) to scores of personalized painful stimuli (NCS-R-PS). A secondary aim of the study was to verify possible correlations between the NCS-R-PS and Coma Recovery Scale—Revised (CRS-R) and to estimate convergent validity. **Methods:** Sixty-one patients with prolonged DoCs (pDoCs) were enrolled from seven European post-acute rehabilitation centers. Responsiveness and pain perception were assessed by CRS-R and NCS-R with standard stimulus (NCS-R-SS) and personalized stimulation (NCS-R-PS). ClinicalTrials.gov Identifier: NCT06012357. **Results:** our results support our prior findings on the superiority and the validity of the personalized painful stimulus approach in assessment of pain in persons with DoCs in comparison with the standardized pain assessment methodology. **Conclusions**: A more in-depth and tailored assessment of pain perception in persons with a DoC may lead to better acknowledgment of its presence and by extension an objective foundation for more aggressive and appropriate pain management.

## 1. Introduction

The assessment, management and treatment of pain in persons with a prolonged disorder of consciousness (pDoC) [1] remains a matter of debate. Functional communication impairments in patients with Vegetative State (VS)/Unresponsive Wakefulness Syndrome (UWS) and Minimally Conscious State (MCS) makes assessment of pain a challenging task [2,3,4]. Persons with pDoCs may present with different sources of pain, either centrally (spasticity, thalamic pain, dystonia, etc.) or peripherally (invasive devices, pressure ulcers, fractures, contractures, neurogenic heterotopic/periarticular ossification, etc.) mediated. These patients may also demonstrate sensory perception abnormalities such as hyperesthesia, hypoesthesia, anesthesia or allodynia, which may confound and/or interfere with interpretation of behavioral responses to pain stimuli [5,6,7].

**Figure 1:**
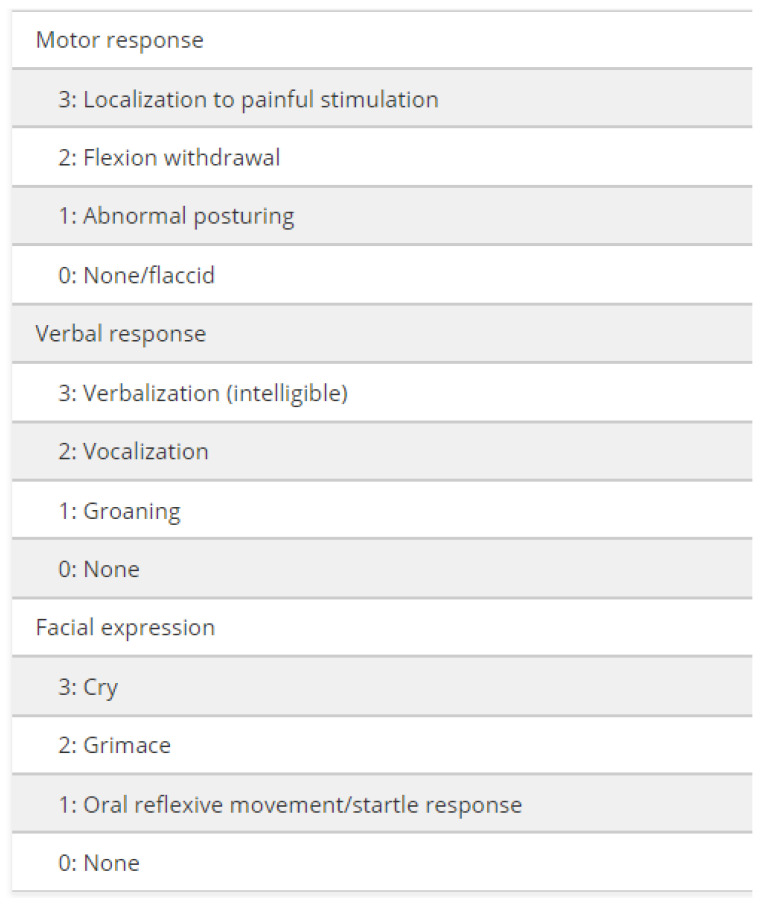
Items assessed by the Nociception Coma Scale—Revised from Chatelle, C., Majerus, S., Whyte, J., Laureys, S. and Schnakers, C. (2012). A sensitive scale to assess nociceptive pain in patients with disorders of consciousness. *Journal of Neurology, Neurosurgery & Psychiatry*, 83 (12), 1233–1237 [10].

The Nociception Coma Scale (NCS) [8] has been specifically developed to assess (motor, verbal, facial and visual) behaviors in response to pain stimulation in patients with pDoCs and disentangle reflex from higher-level behaviors in response to external stimulation, including painful stimuli [9]. A revised version (NCS-R) [10] has been proposed, without the visual subscale, since this latter sub score was not found to be sensitive to the detection of noxious stimulation in these patients (Figure 1).

Both the NCS-R and the NCS have nevertheless been initially validated with experimental pain (i.e., pressure on the fingernail) instead of clinical pain, which might limit their relevance in a clinical setting. In a prior preliminary study, we found that NCS-R behavioral responses to personalized painful stimulation (NCS-R-PS; clinical pain) led to higher scores in patients with pDoCs compared to NCS-R responses to standard stimuli (NCS-R-SS; experimental pain) [11]. This suggests that determining an individual pain threshold using clinical pain might be more relevant than using a one-size-fits-all experimental pain. The main aim of the present study was to confirm (or not) these preliminary results and compare the NCS-R scores obtained with the standard pressure on fingernail bed (NCS-R-SS) to those obtained following personalized painful stimuli (NCS-R-PS), as observed in a large cohort of patients by professionals and caregivers who were involved in this international multicentric project. A secondary aim of the study was to assess the relation between the NCS-R-PS and the Coma Recovery Scale—Revised (CRS-R) and to estimate convergent validity. The CRS-R is indeed the gold standard of responsiveness assessment according to the American Academy of Neurology and the European guidelines for coma and pDoC diagnosis.

## 2. Materials and Methods

Sixty-one patients were enrolled in this study from among the following 7 European post-acute rehabilitation centers: Santa Lucia Foundation, Scientific Institute for Research and Health Care; IRCCS Fondazione Don Carlo Gnocchi, (Firenze and Sant′Angelo dei Lombardi); Coma Science Group, GIGA-Consciousness, University of Liège; Neurological and Internal Medicine Service “Santa Viola”, Hospital (Bologna); and IRENEA Instituto de Rehabilitación Neurológica, Fundación Hospitales Vithas (Valencia and Sevilla).

The study was approved on 9th July 2019 by local ethical committee of the coordinator group (IRCCS Santa Lucia Foundation) with number CE/PROG.603.

All consecutively admitted patients were screened from July 2019 to December 2023 with the following inclusion criteria: i. severe Acquired Brain Injury (sABI), documented by clinical history (i.e., Glasgow Coma Scale (GCS), score ≤8 lasting at least 24 h after brain injury; [12]) and neuroimaging (brain CT and/or MRI) showing focal or diffuse cerebral injury; ii. diagnosis of pDoC (VS/UWS or MCS), according to the Coma Recovery Scale—Revised (CRS-R) [3] as per its validated versions [3,13,14,15], repeated at least 5 times on different days and at rehabilitation admission (best score) [16]; iii. age ranging between 18 and 65 years. Exclusion criteria included a history of previous brain injury, other neurological diagnoses, psychiatric disorder, alcohol or illicit drug abuse and/or spinal cord injury.

The revised version of the NCS (NCS-R) was used [10,17]. In its current version, the NCS-R includes 3 subscales assessing motor, verbal and facial expression responses to standardized nociceptive stimuli (i.e., pressure on fingernail bed), with a total score ranging from 0 to 9. In order to determine the NCS-R-PS, caregivers (including relatives, nurses, therapists and physicians) were asked to record all stimuli which induced a patient′s reaction related to unpleasant conditions. The stimulus that was reported at least by 2 members of the rehabilitation staff (or by one of them and one caregiver) and that consistently induced the highest NCS-R scores was chosen as the personalized stimulus (PS). The NCS-R-PS was determined within 30 days after admission and compared with the standard stimulus (NCS-R-SS), choosing the highest score as reference at admission, 1 month and 3 months after admission, until recovery of consciousness (and emergence from pDoC) or discharge. When no personalized stimuli were identified, the standard stimulus of the validated NCS-R was used.

The CRS-R was recorded in parallel to the NCS-R-SS and NCS-R-PS. According to the CRS-R manual administration guidelines, the patient was observed and evaluated for 5 min, at rest, without any visual, acoustic and tactile stimulation. Spontaneous movements/facial expressions were then recorded and excluded as possible responses/reactions to standard or personalized painful stimuli. The NCR-R-SS and NCR-R-PS were blindly recorded by two different therapists for each center with expertise in DoCs and CRS-R assessment.

The NCS-R with standard stimulus (NCS-R-SS) and with personalized stimulus (NCS-R-PS) were recorded 30 days after admission (T0). Both the CRS-R and NCS-R (SS and PS) were repeated after 1 month (T1) and after 3 months (T2) from T0 utilizing the same procedures as noted above.

### Statistical Analysis

Sample size was computed on the basis of the data reported in our previous pilot study [11], and using G-Power 3.1 software, setting alfa level at 5% and power of analysis at 80%, with an effect size of 0.33, the minimum number of patients required for this study was 61 patients (for the Wilcoxon test).

The Shapiro–Wilk test, performed with a Kolgomorov–Smirnov adjustment for sample > 50, was used to assess the normality of data distribution, and because the *p*-value was <0.05, non-parametric tests were used. Concurrent validity was assessed using the Spearman’s correlation coefficient (R) computed between the scores of the NCS-R-PS and the relevant ones of the NCS-R-SS. The hypothesis that the scores were higher for personalized than standardized stimuli was tested using the Wilcoxon rank test, hypothesizing a positive mean difference (MD = PS score–SS score > 0, *p* < 0.05) corresponding to a higher sensitivity. The correlation between the total scores of the NCS-R-PS (and those of NCS-R-SS), averaged between the two operators, with the total scores of the CRS-R (highest score among 5 assessments) [16] was computed to test the convergent validity of the NCS-R-PS. The item reliability was tested intra-operator using Cronbach’s alpha and considered unacceptable if <0.6, questionable between 0.6 and 0.7, acceptable if >0.7 and good if >0.8 [18]. The inter-operator reliability was tested by analyzing the correlation R between two operators for the NCS-R-PS scores and also for those of the NCS-R-SS and hypothesizing the absence of a difference between operators (Wilcoxon rank test, hypothesis: *p* > 0.05 and a small absolute mean difference, ABS). The threshold of statistical significance was set at alpha = 0.05 for all tests utilized in the statistical analysis.

## 3. Results

### 3.1. Sample Characteristics

The 61 participants with pDoC enrolled had a mean age of 48.4 ± 19.4 years; forty (40) were males and twenty-one (21) were females. Mean time between the acute event and the admission to the neurorehabilitation ward was 102 ± 85 days. The diagnosis was MCS for 36 patients (59%) and VS/UWS for the other 25 (41%). Of the 61 patients assessed at baseline, 46 were assessed again after one month (T1, drop-out rate: 25%) and 38 after three months (T2, drop-out rate: 38%).

DoC etiologies included vascular insults (*n* = 24; 39%), traumatic brain injury (*n* = 20; 33%), anoxic–hypoxic (*n* = 10; 16%), mixed (*n* = 4; 7%) and other (*n* = 3; 5%). Personalized painful stimuli (NCS-R-PS) frequency is reported in Table 1.

### 3.2. Concurrent and Convergent Validity

The primary aim of the study was satisfied. Indeed, the validity of our hypothesis was confirmed as the scores of the NCS-R-PS were significantly correlated with those of the NCS-R-SS (concurrent validity) and those of the CRS-R (convergent validity).

The total scores were computed for each assessment timing averaging those of two operators for NCS-R scales and using the maximum score of five assessments for the CRS-R. Table 2 showed that all these correlations were statistically significant at baseline, after one month (T1) and after 3 months (T2).

When single scores were analyzed, the concurrent validity should be verified by significant correlations between NCS-R-PS and NCS-R-SS for each single experimenter at each single assessment. To confirm the hypothesis that NCS-R-PS was more sensitive than NCS-R-SS, the mean difference (MD) computed for each score had to be statistically significant (*p* < 0.05) and positive (MD > 0), as shown in Table 3. All the correlations were statistically significant and all the MDs were positive. In particular, the MDs ranged between 1 and 1.5, always positive, meaning higher scores for personalized than for standardized stimuli. The only not statistically significant MDs in Table 3 were the motor score for experimenter 2 at baseline (MD = 1, *p* = 0.066), verbal score after 1 month for both the experimenters (MD = 1, *p* = 0.137 and *p* = 0.107, respectively) and the scores assessed after 3 months. The secondary aim of the study was also confirmed. Indeed, a correlation with the CRS-R was found for both the NCS-SS and NCS-PS (Table 2).

### 3.3. Intra-Operator Reliability

The range of Cronbach’s alpha, for both the experimenters, was acceptable (>0.7) or good (>0.8) at baseline and T1, whereas it was questionable only at T2, as shown in Table 4. However, the average of Cronbach’s alpha for personalized stimuli was slightly superior to that of standard stimuli (0.737 vs. 0.724). The average values of Cronbach’s alpha were acceptable both for personalized and standard stimuli.

### 3.4. Inter-Operator Reliability

To assess the inter-operator reliability, the correlation between the scores given by the two operators at the same timing in terms of NCS-R-PS or NCS-R-SS was computed and reported in Table 5. All these correlations were highly statistically significant both for personalized and standard stimuli (*p* < 0.001). It is noteworthy that the Spearman’s correlation coefficients between the scores assigned by the two operators were higher for all the three sub-items and in all the three timings for personalized stimuli, with the only exception of motor response after one month. Neither for personalized nor for standard stimuli were the differences between the two experimenters statistically significant. The range of absolute mean difference was between 0 and 2, but not statistically significant.

## 4. Discussion

The results of this international multicenter study support our prior findings on the superiority of the personalized painful stimulus approach in assessment of pain in persons with DoCs in comparison with the standardized pain assessment methodology [11]. The validity of both the NCS-R-SS and NCS-R-PS scales was demonstrated by the correlations with the relevant scores assessed using standardized stimuli and with the total score of the CRS-R. Then, NCS-R-PS scores were equal to or higher than those obtained with standardized stimuli, and not negative in any case. The mean difference ranged between 0.5 and 1.5 points more for the NCS-R-PS than for the NCS-R-SS. Especially at the beginning of rehabilitation and within 1 month, these differences were found to be statistically significant, suggesting the importance of using personalized stimuli especially at the beginning of the rehabilitation pathway.

The personalization of the stimuli, in comparison to the use of standard stimuli, could be more sensitive. One would argue it could also increase the variability and reduce the reliability, since it is not standardized. However, this does not seem to be the case based on our results, since good (intra and inter) operator reliability was obtained. The reduced reliability at T2 could be due to a combination of floor/ceiling effects and/or the reduction in the sample size because of participants’ drop out. Actually, a lower reliability at T2 was observed for both standardized and personalized stimuli, suggesting the impact of experimental bias (change in number of raters across time) instead of poor psychometric properties.

It should be noted as, for most of clinical scales, floor/ceiling effects may reduce the sensitivity of the scale. In our case, the NCS-R-SS seemed to be affected by a floor effect (with many scores = 0) and it can be the reason for the higher sensitivity of NCS-R-PS.

Of note, the heterogeneity of the neurological deficits in patients with pDoCs as well as pathologic pain responses (such as allodynia) may cause a lack or reduction in responsivity to standard painful stimuli [7]. Indeed, if one patient has hypoesthesia in one part of the body, he could show no response with the standardized painful stimulus, whereas he could present behavioral responses with a personalized painful stimulus. Ultimately, the choice of a personalized and tailored painful stimulus for use in pain assessment should be guided by behavioral observations of possible pain-inducing stimuli during nursing or physiotherapy. Among the pain generators, muscle tone disorders, prolonged bed-rest syndrome, skin pressure ulcers, external devices such as tubes and urinary catheters as well as neurogenic heterotopic ossification have been reported [5,15,19,20,21,22,23]. Central/thalamic or neuropathic pain may represent further sources of pain in patients with DoCs, and thus an individualized pain assessment should be advocated for [7,24,25,26].

The inclusion of caregivers in the bedside behavioral assessment of persons with pDoCs should be encouraged as it could lead to earlier identification of signs of pain perception in their loved one. This has been shown in other contexts such as during consciousness assessment [27].

Moreover, lower pain pressure thresholds have been reported in persons with DoCs in comparison with healthy controls, and induced indirect pain indicators are frequently observed by nurses, physiotherapists or caregivers, consisting of mimic reactions, grimacing, crying or shouting, vocalization and verbalization [28]. Psychomotor agitation, in a resting state or during nursing maneuvers or physiotherapy, is also commonly reported in non-communicative patients [17,23,24,25,29].

The major strength of the present study is based on the international multicenter data collection and the confirmation of the superiority of a patient-centered, multidisciplinary approach in the assessment of pain perception in persons with DoCs versus standardized painful stimulus, regardless of professional staff and caregivers’ indications. These results support other studies noting the importance of using customized stimuli in the assessment of persons with pDoCs in order to increase response likelihood [30,31].

Potential study limitations included the following: (i) the lack of data on the influence of pain medications and/or prophylactic pain management interventions on pain stimuli response [7,32,33]; (ii) the participation of several researchers because of the possible inhomogeneity of the different behavioral assessments of the raters; and (iii) the different languages/cultures involved. Nonetheless, these limitations should have influenced the results of both the pain assessment methods using either standard or personalized pain stimuli.

## 5. Conclusions

The higher sensitivity of the NCS-R-PP supports the preferential use of personalized pain stimuli in the assessment of pain perception in those patients with DoCs, especially when the NCS-R-SS does not generate significant behavioral responses in such patients. Furthermore, an individualized painful stimulus may unmask behavioral reactions to pain in persons diagnosed with covert cognition or cognitive–motor dissociation [34,35]. The strong positive correlation of the NCS-R-PS with the CRS-R seems to confirm previous data on the parallel evolution of NCS-R and CRS-R scores [36,37]. Ethical considerations on the use of this pain assessment tool include avoiding the additional painful maneuvers of the standard pain stimuli, as well as the possibility to offer more detailed and individualized feedback to involved parties (clinicians, family, caregivers, lawyers, etc.) on the implications of assessment findings on the presence versus absence of pain perception and, by potential implication, suffering [7]. In conclusion, a more in-depth and tailored assessment of pain perception in persons with a DoC may lead to better acknowledgment of its presence and by extension an objective foundation for more aggressive and appropriate pain management.

## Figures and Tables

**Table 1:** Frequency of the personalized painful stimuli.

BODY PART USED FOR THE PERSONALIZED PAIN FUL STIMULI	NUMBER OF PATIENTS
LEFT HAND OPENING	8
NIPPLES SQUEEZING	4
RIGHT HAND OPENING	4
RIGHT THUMB MOBILIZATION	4
RIGHT HAND CLOSURE	3
LEFT UPPER LIMB FLEXION	2
RIGHT UPPER LIMB EXTENSION	2
RIGHT KNEE EXTENSION	2
RIGHT UPPER LIMB ABDUCTION	1
RIGHT LOWER LIMB EXTENSION	1
RIGHT ELBOW EXTENSION	1
LEFT ELBOW EXTENSION	1
RIGHT HIP EXTENSION	1
LOWER LIMB EXTENSION	1
RIGHT FINGERS FOOT FLEXION	1
MOBILIZATION IN SITTING POSITION	1
RIGHT LOWER LIMB MOBILIZATION	1
LEFT LOWER LIMB MOBILIZATION	1
RIGHT LOWER LIMB MOBILIZATION	1
RIGHT HEAD ROTATION	1
RIGHT UPPER LIMB MOBILIZATION (BONE FRACTURE)	1
LEFT FOOT MOBILIZATION	1
SHOULDER MOBILIZATION	1
RIGHT EAR PRESSURE (BEDSORE)	1
CALF PRESSURE	1
TRAPEZIUS MUSCLE PRESSURE	1
RIGHT HIP INTRAROTATION	1

**Table 2:** Spearman′s correlation coefficient R and relevant *p*-values among the NCS-R-PS, NCS-R-SS and CRS-R total scores at baseline, after one month (T1) and after three months (T2) for the *N* patients.

Spearman Correlation	Baseline	T1	T2
	*N* = 61	*N* = 46	*N* = 38
NCS-R-PS vs. NCS-R-SS	R = 0.555 *p* < 0.001	R = 0.750 *p* < 0.001	R = 0.724 *p* < 0.001
NCS-R-PS vs. CRS-R	R = 0.388 *p* = 0.002	R = 0.498 *p* < 0.001	R = 0.549 *p* < 0.001
NCS-R-SS vs. CRS-R	R = 0.484 *p* < 0.001	R = 0.416 *p* = 0.004	R = 0.351 *p* = 0.028

**Table 3:** Spearman′s correlation coefficients (R) computed between scores of the NCS-R-PS and NCS-R-SS and mean differences (MD) for the two experimenters. * *p* < 0.05.

Assessment Timing	NCS-R score	Spearman Correlation Coefficient		Mean Difference PS—SS	
		Experimenter 1	Experimenter 2	Experimenter 1	Experimenter 2
**Baseline**	**Motor**	R = 0.520 *	R = 0.428 *	MD = 1 *	MD = 1
	**Verbal**	R = 0.814 *	R = 0.845 *	MD = 1 *	MD = 1 *
	**Facial**	R = 0.590 *	R = 0.556 *	MD = 1.5 *	MD = 1 *
**After 1 month**	**Motor**	R = 0.571 *	R = 0.654 *	MD = 1 *	MD = 1 *
	**Verbal**	R = 0.776 *	R = 0.779 *	MD = 1	MD = 1
	**Facial**	R = 0.618 *	R = 0.622 *	MD = 1*	MD = 1.5 *
**After 3 months**	**Motor**	R = 0.417 *	R = 0.353 *	MD = 1	MD = 0.5
	**Verbal**	R = 0.670 *	R = 0.580 *	MD = 1	MD = 1
	**Facial**	R = 0.615 *	R = 0.602 *	MD = 1	MD = 1

**Table 4:** Cronbach′s alpha values for NCS-R-PS and NCS-R-SS, for the two experimenters at the three assessment timings. The last column reports the mean ± standard deviation of the Cronbach’s alpha values among experimenters and timings.

Cronbach′s Alpha	Experimenter 1			Experimenter 2			Mean ± SD
	Baseline	T1	T2	Baseline	T1	T2	
**NCS-R-PS**	0.771	0.783	0.630	0.801	0.808	0.630	0.737 ± 0.084
**NCS-R-SS**	0.815	0.821	0.554	0.779	0.851	0.522	0.724 ± 0.146

**Table 5:** The Spearman correlation coefficient (R) and the relevant absolute mean difference (AMD) between the two operators for NCS-R-PS and NCS-R-SS motor, verbal and facial expression scores at the three assessment timings. *p < 0.001.

Assessment Timing	NCS-R Score	Spearman Correlation Coefficient		Absolute Mean Difference	
		NCS-R-PS	NCS-R-SS	NCS-R-PS	NCS-R-SS
**Baseline**	**Motor**	R = 0.884 *	R = 0.664 *	AMD = 0	AMD = 0
	**Verbal**	R = 0.977 *	R = 0.975 *	AMD = 0	AMD = 0
	**Facial**	R = 0.910 *	R = 0.812 *	AMD = 1.47	AMD = 1
**After 1 month**	**Motor**	R = 0.889 *	R = 0.974 *	AMD = 0.5	AMD = 1
	**Verbal**	R = 0.998 *	R = 0.997 *	AMD = 0	AMD = 0
	**Facial**	R = 0.925 *	R = 0.872 *	AMD = 0.2	AMD = 1
**After 3 months**	**Motor**	R = 0.946 *	R = 0.884 *	AMD = 2	AMD = 1
	**Verbal**	R = 0.999 *	R = 0.910 *	AMD = 0	AMD = 0.1
	**Facial**	R = 0.862 *	R = 0.847 *	AMD = 0	AMD = 0.1

* *p* < 0.05.

## Data Availability

The datasets presented in this article are not available in order to respect patients’/participants’ privacy.
